# Tryptophan Metabolism in Cardiometabolic Diseases: Focus on the Kynurenine Pathway

**DOI:** 10.3390/ijms27125223

**Published:** 2026-06-09

**Authors:** Shafaat Hussain, Mohamed M. Bekhite, P. Christian Schulze

**Affiliations:** Department of Internal Medicine I, Division of Cardiology, Angiology and Intensive Medical Care, University Hospital Jena, Friedrich-Schiller-University, 07743 Jena, Germany; shafaat.hussain@med.uni-jena.de (S.H.); mohamed.el_saied@med.uni-jena.de (M.M.B.)

**Keywords:** tryptophan metabolism, kynurenine pathway, cardiometabolic diseases, metabolomics, inflammation, oxidative stress

## Abstract

Tryptophan (TRP) metabolism has emerged as a critical interface linking inflammation, immune regulation, oxidative stress, and cellular energetics. The kynurenine pathway, the predominant route of TRP degradation, is highly responsive to inflammatory stimuli and generates a spectrum of bioactive metabolites with divergent and context-dependent biological effects. Indoleamine 2,3-dioxygenase 1 (IDO1)-mediated TRP catabolism integrates immune activation with downstream metabolic signaling, influencing redox homeostasis, endothelial function, and mitochondrial energetics, in part by regulating nicotinamide adenine dinucleotide (NAD^+^) synthesis. Alterations in TRP metabolism are consistently observed across cardiometabolic diseases, including obesity, type 2 diabetes (T2D), atherosclerosis, myocardial infarction (MI), and heart failure with preserved ejection fraction (HFpEF), where they are associated with disease severity and adverse outcomes. Importantly, emerging data suggest that cardiometabolic phenotypes are determined not by pathway activation alone, but by the relative distribution of flux across downstream metabolic branches. Depending on the tissue compartment and stage of the disease, different biological effects may be contributed by redox-active kynurenine 3-monooxygenase (KMO)/3-hydroxykynurenine (3-HK)/quinolinic acid (QA) pathways, 3-hydroxyanthranilic acid (3-HAA)-mediated lipid and inflammasome regulation, microbiome-derived indoles, and NAD^+^-generating pathways. This review synthesizes current evidence using a branch-specific and context-dependent framework. We discuss the utility and limitations of the kynurenine-to-tryptophan ratio (KTR) as an upstream biomarker, the need for downstream metabolite panels, and therapeutic opportunities aimed at pathway modulation rather than broad inhibition. Future studies integrating temporal profiling, spatial and cell-specific approaches, large-animal models, and pathway-informed clinical trials will be essential to define causal mechanisms and enable precision therapeutic translation.

## 1. Introduction

Chronic inflammation and metabolic dysregulation are central and tightly interconnected drivers of cardiometabolic diseases [1,2,3]. Increasing evidence indicates that inflammatory signaling actively reshapes cellular metabolism, thereby influencing redox balance, mitochondrial function, and tissue homeostasis. In this context, metabolic pathways act not only as downstream targets of inflammation but also as key mediators linking immune activation to organ dysfunction [1,2,3].

Among these pathways, tryptophan (TRP) metabolism has emerged as an important interface between immune signaling and metabolic regulation [1,2,3]. TRP is an essential amino acid obtained through dietary intake and serves as a precursor for several biologically active compounds. While a portion of TRP is utilized for protein synthesis or metabolized via serotonin and microbiome-dependent pathways, the majority is degraded through the kynurenine pathway, which represents the predominant route of TRP catabolism [1,3,4].

The kynurenine pathway is highly responsive to inflammatory stimuli and generates a range of intermediate metabolites, collectively referred to as kynurenines (KYNs), that exert diverse biological effects across immune and metabolic processes [1,3,4]. A central feature of this pathway is its regulation by inflammatory signaling. The rate-limiting enzyme indoleamine 2,3-dioxygenase 1 (IDO1) is inducible by pro-inflammatory cytokines, thereby linking TRP degradation directly to immune activation and downstream metabolic responses [3].

Experimental studies indicate that kynurenine pathway metabolites influence endothelial function, vascular biology, and myocardial metabolism [5,6,7,8]. Consistent with these observations, alterations in circulating TRP metabolites have been reported across cardiometabolic diseases, including obesity, diabetes, atherosclerosis, myocardial infarction (MI), and heart failure (HF) [1,2,9,10,11,12,13,14,15,16,17,18,19,20,21,22,23,24,25,26,27,28,29,30,31,32,33,34,35,36,37,38,39,40,41,42,43,44,45,46,47,48,49,50,51,52]. Among these conditions, heart failure with preserved ejection fraction (HFpEF) has been consistently associated with changes in TRP metabolism in metabolomic studies, although the extent to which these alterations contribute to disease mechanisms remains unclear [42,43,44,45,46,47,48,49,50,51,52].

Previous reviews have summarized the broad role of TRP metabolism in cardiometabolic diseases and the relationship between the kynurenine pathway, carbohydrate metabolism, diabetes, and cardiovascular risk [53,54]. However, a remaining challenge is that kynurenine pathway activation is often interpreted as a single inflammatory signal, even though its downstream metabolites can have divergent and even opposing biological effects [6,19,55,56]. These effects may vary according to tissue compartment, inflammatory or metabolic state, disease stage, and downstream metabolic branch [6,19,55,56]. The present review therefore, aims to extend prior work by organizing current evidence around a branch-specific and context-dependent framework for cardiometabolic diseases. Rather than focusing only on overall pathway activation, we emphasize how distinct TRP metabolic routes may shape vascular inflammation, myocardial energetics, adipose tissue dysfunction, gut barrier regulation, and immune responses. We further discuss how upstream biomarkers, downstream metabolite panels, and pathway-directed therapeutic strategies may support a transition from association-based observations toward mechanism-informed risk stratification and intervention.

## 2. Tryptophan Metabolism and the Kynurenine Pathway

**Figure 1:**
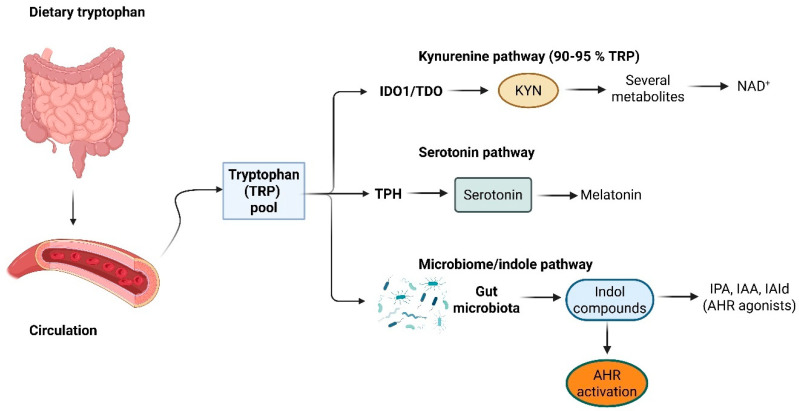
**Overview of tryptophan (TRP) metabolism pathways.** Dietary TRP is absorbed in the intestine and enters the systemic circulation, contributing to the circulating TRP pool available for cellular uptake. TRP is metabolized through three major pathways. The kynurenine pathway, accounting for approximately 90–95% of TRP degradation, is initiated by indoleamine 2,3-dioxygenase 1 (IDO1) or tryptophan-2,3-dioxygenase (TDO), leading to the formation of kynurenine (KYN) and downstream metabolites that ultimately contribute to nicotinamide adenine dinucleotide (NAD^+^) synthesis. The serotonin pathway, mediated by tryptophan hydroxylase (TPH), converts TRP into serotonin, which can be further metabolized into melatonin. In parallel, TRP is metabolized by the gut microbiota into indole-derived compounds, including indole-3-propionic acid (IPA), indole-3-acetic acid (IAA), and indole-3-aldehyde (IAld), which act as ligands for the aryl hydrocarbon receptor (AHR) and modulate host signaling pathways.

TRP is an essential aromatic amino acid obtained exclusively through dietary intake and plays fundamental roles in protein synthesis and cellular metabolism [3]. Major dietary sources of TRP include protein-rich foods such as poultry, fish, eggs, milk and dairy products, soybeans and soy products, legumes, oats, peanuts, pumpkin seeds, sesame seeds, and sunflower seeds [57]. In addition to its incorporation into proteins, TRP serves as a precursor for several biologically active metabolites that regulate immune responses, oxidative stress, and cellular signaling pathways [3]. Only a small proportion of TRP is used for protein synthesis, whereas the majority undergoes enzymatic degradation through specialized metabolic pathways (Figure 1) [1,3,4]. Three principal metabolic routes of TRP generation have been described: the serotonin pathway, microbial metabolism mediated by the intestinal microbiota, and the kynurenine pathway (Figure 1) [1,3,4]. Among these pathways, the kynurenine pathway represents the dominant route of TRP degradation in mammals, accounting for approximately 90–95% of TRP catabolism (Figure 1) [1,3,4]. The kynurenine pathway generates a diverse array of metabolites collectively referred to as KYNs, which exert important biological effects on immune regulation, redox homeostasis, and cellular metabolism [1,3,4].

### 2.1. Tryptophan Metabolic Pathways

Following intestinal absorption, dietary TRP enters the systemic circulation and is transported to various tissues where it participates in diverse metabolic processes. Cellular uptake of TRP is mediated by specific transporters, including solute carrier family 1 member 5 (SLC1A5) and solute carrier family 7 member 5 (SLC7A5), which facilitate its entry into cells and distribution to organs such as the brain, heart, and skeletal muscle [58]. These processes involve both host enzymatic pathways and microbial metabolism within the gastrointestinal tract [58]. One well-characterized route of TRP metabolism is the serotonin pathway, which converts TRP into serotonin (5-hydroxytryptamine) through sequential enzymatic reactions catalyzed by tryptophan hydroxylase (TPH) and aromatic L-amino acid decarboxylase (ALAAD) [3,59]. Serotonin acts as an important neurotransmitter regulating mood, appetite, gastrointestinal motility, and vascular tone [60,61]. Subsequent metabolism of serotonin leads to the synthesis of melatonin, a hormone that plays a key role in circadian rhythm regulation and sleep–wake cycles (Figure 1) [62]. In addition to host metabolic pathways, TRP can also be metabolized by intestinal microbiota into a variety of indole derivatives, including indole-3-propionic acid (IPA), indole-3-acetic acid (IAA), and indole-3-aldehyde (IAld) (Figure 1) [63]. These microbiota-derived metabolites interact with host signaling pathways such as the aryl hydrocarbon receptor (AHR) [63], and contribute to the regulation of intestinal barrier integrity, immune responses, and host–microbiome communication (Figure 1) [1]. Alterations in gut microbial composition can therefore influence systemic TRP metabolism and contribute to inflammatory and metabolic disorders [1].

Despite the biological importance of these alternative pathways, the vast majority of TRP degradation occurs through the kynurenine pathway (Figure 1) [63]. Within this pathway, TRP is converted through a series of intermediate metabolites that ultimately lead to the synthesis of nicotinamide adenine dinucleotide (NAD^+^) (Figure 1) [63]. Because NAD^+^ is a critical cofactor involved in mitochondrial respiration, redox reactions, and cellular energy metabolism, the kynurenine pathway represents an important metabolic link between amino acid metabolism and cellular energetics [64].

### 2.2. Enzymatic Cascade and Regulation of Kynurenine Pathway

**Figure 2:**
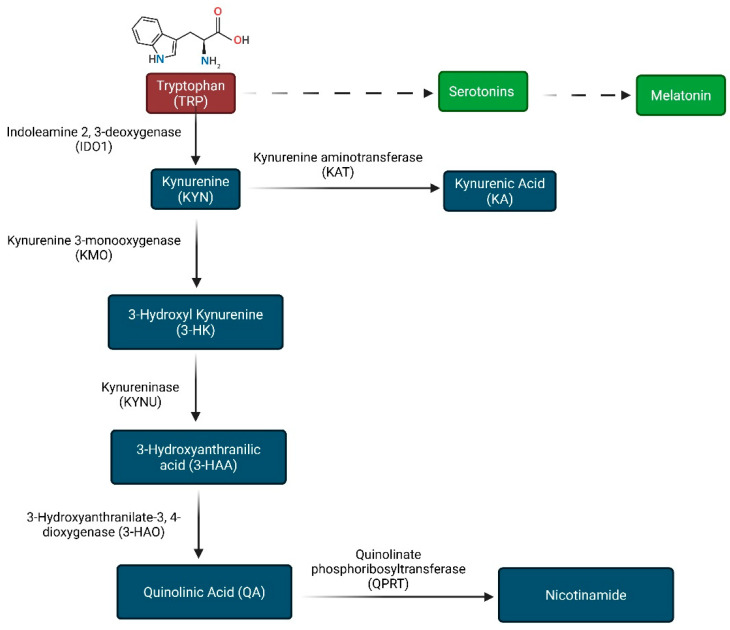
**Overview of the tryptophan (TRP)–kynurenine pathway and its major metabolic branches.** This figure illustrates the principal metabolic pathways of TRP. TRP can be converted into serotonin and subsequently melatonin. Alternatively, TRP is metabolized by indoleamine 2,3-dioxygenase (IDO1) to form kynurenine (KYN), initiating the kynurenine pathway. KYN is further metabolized through two main branches. Via kynurenine aminotransferase (KAT), KYN is converted to kynurenic acid (KA). Through kynurenine 3-monooxygenase (KMO), KYN is converted to 3-hydroxykynurenine (3-HK), which is subsequently metabolized by kynureninase to 3-hydroxyanthranilic acid (3-HAA). 3-HAA is further converted by 3-hydroxyanthranilate 3,4-dioxygenase (3-HAO) to quinolinic acid (QA). Quinolinic acid is then metabolized by quinolinate phosphoribosyltransferase (QPRT) to form nicotinamide.

The kynurenine pathway begins with the oxidative cleavage of the indole ring of TRP to form N-formylkynurenine, which is rapidly converted into kynurenine (KYN) (Figure 2) [1,3]. This rate-limiting step is catalyzed by two enzymes: tryptophan-2,3-dioxygenase (TDO) and IDO1 [1,3]. TDO is primarily expressed in hepatocytes and plays an important role in maintaining systemic TRP homeostasis under physiological conditions [65]. Its activity is largely regulated by substrate availability and hormonal signals such as glucocorticoids [65]. In contrast, IDO1 is expressed in a wide range of extrahepatic tissues, including immune cells, epithelial cells, and vascular endothelial cells. Unlike TDO, IDO1 is strongly inducible and is activated by inflammatory mediators such as interferon-γ (IFN-γ), tumor necrosis factor-α (TNF-α), lipopolysaccharide (LPS), and reactive oxygen species (ROS) [66,67]. Following the formation of KYN, the pathway diverges into several downstream branches that generate distinct metabolites with diverse biological properties. One branch converts KYN into kynurenic acid (KA) through the activity of KYN aminotransferases (KATs) (Figure 2) [3]. KA has been described as a neuroactive metabolite and has been reported to exert anti-inflammatory effects in certain biological contexts [3]. Another important branch involves the conversion of KYN into 3-hydroxykynurenine (3-HK) by kynurenine-3-monooxygenase (KMO) (Figure 2). This metabolite is redox-active and has been associated with the generation of ROS and oxidative stress [3]. Downstream metabolism of 3-HK by kynureninase (KYNU) results in the formation of 3-hydroxyanthranilic acid (3-HAA). This intermediate is subsequently converted by 3-hydroxyanthranilate dioxygenase (3-HAO) into quinolinic acid (QA), which represents a key precursor for the de novo synthesis of NAD^+^ (Figure 2) [68]. Through this sequence of reactions, the kynurenine pathway links TRP degradation to mitochondrial metabolism and cellular energy homeostasis [69].

The activity of the kynurenine pathway is tightly regulated by inflammatory, metabolic, and environmental signals. Among these regulatory mechanisms, induction of IDO1 by pro-inflammatory cytokines represents the most important driver of pathway activation [3,70]. IFN-γ is considered the most potent inducer of IDO1 expression. Activation of IFN-γ signaling stimulates transcription of the IDO1 gene through the Janus kinase–signal transducer and activator of transcription (JAK–STAT) pathway, leading to accelerated degradation of TRP and accumulation of KYN metabolites [70]. Additional inflammatory mediators, including TNF-α, interleukin-1β (IL-1β), and bacterial LPS, can further amplify this response [71,72]. Oxidative stress also contributes to regulation of TRP metabolism [73]. ROS and angiotensin II (Ang II) have been shown to induce IDO1 expression in endothelial cells, linking activation of the kynurenine pathway to vascular inflammation and endothelial dysfunction [6]. Another important regulatory mechanism involves substrate availability. Depletion of TRP within the cellular microenvironment can suppress proliferation of activated T lymphocytes and promote immune tolerance. These mechanisms highlight the close relationship between TRP metabolism and immune regulation [74].

### 2.3. Branch-Specific and Context-Dependent Effects of Kynurenine Pathway Metabolites

A critical and often underappreciated feature of the kynurenine pathway is that the metabolic branch selected downstream of KYN can shape divergent biological outcomes. The KAT branch, which converts KYN into KA, has been associated with regulatory and cytoprotective effects in selected contexts [18,19,55,56,75,76]. KA acts as a ligand for G protein-coupled receptor 35 (GPR35), which in adipose tissue has been linked to improved energy homeostasis and reduced inflammation [18,76]. In endothelial cells, KA can activate peroxisome proliferator-activated receptor delta (PPARδ) and heme oxygenase-1 (HO-1) signaling to limit inflammation, oxidative stress, and apoptosis [55]. In atherosclerosis, KA also signals through AHR in activated human macrophages, reducing TNF-α, interleukin-6 (IL-6), C-C chemokine receptor 7 (CCR7), C-C motif chemokine ligand 5 (CCL5), and C-X-C motif chemokine ligand 10 (CXCL10), supporting an anti-inflammatory role within the vascular wall [19].

In contrast, the KMO/KYNU branch produces 3-HK, 3-HAA, and ultimately QA, linking inflammatory TRP metabolism to redox signaling, lipid metabolism, and de novo NAD^+^ synthesis [3,4,6,68,69]. 3-HK is redox-active and can promote ROS generation through NADPH oxidase activation, thereby contributing to endothelial apoptosis and dysfunction [6]. By contrast, 3-HAA has been reported to suppress inflammasome activation, reduce interleukin-1β (IL-1β) production, modulate lipid metabolism, and limit low-density lipoprotein (LDL) oxidation and oxidized LDL (oxLDL) uptake in experimental models [20,22,29]. QA also has a dual role: when efficiently converted through quinolinate phosphoribosyltransferase (QPRT), it serves as an essential precursor for NAD^+^ biosynthesis and may support mitochondrial metabolism and cellular energy homeostasis [68,69,77]. However, when quinolinic-branch activation occurs in a chronically inflamed or oxidizing microenvironment and is not matched by efficient downstream NAD^+^ synthesis, QA and upstream redox-active intermediates may instead indicate maladaptive pathway activation associated with oxidative stress, mitochondrial dysfunction, and tissue injury [3,4,6,50].

Together, these findings indicate that the impact of kynurenine pathway activation depends not only on upstream TRP catabolism, but also on the relative engagement of KAT/KA, KMO/3-HK/3-HAA/QA, and NAD^+^-linked pathways. Experimental evidence supports KA-dependent anti-inflammatory, metabolic, endothelial-protective, and cardioprotective effects in selected settings, whereas chronic inflammatory or metabolic stress may be accompanied by altered KMO/KYNU activity, redox-active metabolite accumulation, or inefficient NAD^+^ metabolic resolution [11,19,20,50,78]. The relevance of this branch-specific framework is considered in the following cardiometabolic disease sections, where different TRP-derived metabolites and signaling pathways appear to predominate depending on disease context.

## 3. Role of Tryptophan Metabolism in Cardiovascular Biology

### 3.1. Endothelial and Vascular Effects

The vascular endothelium plays a central role in maintaining vascular homeostasis by regulating vascular tone, barrier integrity, and inflammatory signaling [79]. Endothelial cells express inducible IDO1, enabling local activation of TRP metabolism in response to inflammatory stimuli [6]. A seminal study by Wang et al. [7] identified KYN as a vasoactive mediator, capable of inducing vascular relaxation through activation of adenylate cyclase and soluble guanylate cyclase pathways [7]. These findings indicate that early activation of the kynurenine pathway may serve an adaptive role in maintaining vascular tone during inflammatory stress. In contrast, a study by Wang et al. demonstrated that downstream metabolites of the kynurenine pathway can have opposing effects. Specifically, 3-HK, produced via KMO, reacts with and chemically modifies the cytosolic nicotinamide adenine dinucleotide phosphate (NADPH) oxidase subunits p47^phox^ and p67^phox^. This modification promotes their translocation to the membrane and subsequent activation of NADPH oxidase, leading to increased intracellular ROS production. The elevated ROS levels trigger mitochondrial cytochrome c release, which in turn activates caspase-9, ultimately inducing endothelial cell apoptosis and contributing to endothelial dysfunction [6]. These observations suggest that sustained or imbalanced kynurenine pathway activation shifts endothelial responses toward oxidative dysfunction. In addition to redox-mediated effects, KYN also functions as a signaling molecule. Molinaro et al. reported that KYN activates the AHR, thereby modulating downstream genes, such as cytochrome p450, family 1, subfamily a, polypeptide 1 (CYP1A1), cytochrome p450, family 1, subfamily b, polypeptide 1 (CYP1B1) expression and exacerbating endothelial-to-mesenchymal transition and osteoblastic differentiation [80]. Following ligand binding, AHR dissociates from its cytosolic chaperone complex, which includes heat shock protein 90 (HSP90), hepatitis B virus X-associated protein 2/aryl hydrocarbon receptor-interacting protein (XAP2/AIP), prostaglandin E synthase 3 (p23), and proto-oncogene tyrosine-protein kinase Src (c-Src), and subsequently translocates to the nucleus where it forms a heterodimer with AHR nuclear translocator (ARNT). The activated AHR/ARNT complex binds xenobiotic response elements (XREs) and induces transcription of several canonical target genes, including CYP1A1, CYP1B1, CYP1A2, aryl hydrocarbon receptor repressor (AHRR), and IDO1. In parallel, AHR can also activate non-canonical signaling pathways involving epidermal growth factor receptor (EGFR), nuclear factor kappa B (NF-κB), retinoblastoma protein (Rb), cellular musculoaponeurotic fibrosarcoma (c-Maf), and Kruppel-like factor 6 (KLF6), which may collectively link KYN signaling to inflammatory activation, oxidative stress, endothelial dysfunction, and vascular remodeling [81].

Furthermore, Lin et al. [82] demonstrated that KYN promotes endothelial cell migration and angiogenesis through activation of the mechanistic target of rapamycin (mTOR) pathway. This activation increases the expression of several pro-angiogenic factors and their receptors, including hypoxia-inducible factor 1-alpha (HIF1A), epidermal growth factor (EGF), epidermal growth factor receptor (EGFR), platelet-derived growth factor subunit B (PDGFB), phosphatase and tensin homolog (PTEN), and angiopoietin-2 (ANG2), further supporting its role in vascular remodeling [82]. In contrast, metabolites produced through KAT activity, such as KA, have been reported to exert anti-inflammatory and potentially antioxidant effects. Lee et al. [55] investigated the effects of KA on LPS-induced inflammation and apoptosis in human umbilical vein endothelial cells and found that KA reduced LPS-induced phosphorylation of NF-κB, decreased the release of pro-inflammatory cytokines, and downregulated adhesion molecule expression. These protective effects were mediated through the upregulation of PPARδ and HO-1, ultimately protecting endothelial cells against inflammation and apoptosis [55], thereby highlighting that the relative activity of KMO versus KAT may represent a critical determinant of vascular outcome.

Recent studies indicate that impaired endothelial IDO1 signaling may contribute to vascular aging through disruption of kynurenine pathway-mediated de novo NAD^+^ synthesis [83]. In peripheral arterial disease, aging-associated reductions in endothelial IDO1 expression decreased NAD^+^ biosynthesis, promoted endothelial senescence, and impaired post-ischemic neovascularization [83]. Mechanistically, elevated interleukin-17A/F (IL-17A/F) signaling suppressed cAMP response element-binding protein (CREB)-dependent IDO1 transcription, whereas restoration of IDO1 activity improved endothelial NAD^+^ metabolism and angiogenic recovery [83].

Overall, these studies demonstrate the complex and context-dependent role of TRP metabolism in vascular biology. Kynurenine pathway metabolites can exert diverse and sometimes opposing effects on endothelial function, ranging from regulation of vascular tone and angiogenesis to promotion of inflammatory signaling, oxidative stress, endothelial senescence, and vascular remodeling. Notably, KYN itself appears capable of mediating both adaptive and detrimental vascular responses depending on the surrounding inflammatory and metabolic environment. These results demonstrate that the relative abundance of downstream metabolites and the signaling pathways they activate within the local milieu, rather than just the level of pathway activation, shape the vascular effects of kynurenine pathway activation.

### 3.2. Myocardial Effects and Metabolic Integration

Beyond the vasculature, TRP metabolism also influences myocardial biology through its integration with cellular energetics, redox signaling, and intercellular communication [1,64]. A central link between the kynurenine pathway and cardiac function is the production of NAD^+^ via QA [77]. NAD^+^ acts as an essential cofactor for mitochondrial respiration and a critical substrate for metabolic regulators that maintain redox homeostasis and energy metabolism [84]. Through its role in activating sirtuin-1 (SIRT1) and enhancing cellular resilience, NAD^+^ has been established as an attractive therapeutic target for cardiovascular diseases [85]. In mammalian cells, NAD^+^ is generated through two principal routes: the salvage pathway, which recycles nicotinamide via the rate-limiting enzyme nicotinamide phosphoribosyltransferase (NAMPT), and the de novo pathway, which converts TRP to NAD^+^ through the kynurenine pathway. Under normal conditions, the salvage pathway supplies most cellular NAD^+^. Supplementing NAD^+^ precursors such as nicotinamide riboside (NR) or nicotinamide mononucleotide (NMN) has been found to delay HF progression in both animal experiments and clinical studies [84,86]. However, the salvage pathway is constrained by NAMPT, which is often reduced in cardiometabolic conditions while being overexpressed in cancers. This limitation highlights the potential therapeutic importance of alternative NAD^+^ biosynthesis pathways. Notably, TRP, the primary substrate of the kynurenine pathway, has demonstrated a greater capacity to support NAD^+^ biosynthesis than nicotinic acid or nicotinamide in mammalian models, suggesting that the de novo pathway may become particularly relevant when salvage pathway function is compromised [87,88]. The dynamic regulation between these two NAD^+^ pathways is highly context-dependent. Experiments using THP-1 human monocytes and peripheral blood mononuclear cells (PBMCs) have demonstrated that cells depend on NAMPT-mediated salvage during low-grade inflammation but switch to IDO1-dependent de novo synthesis during severe sepsis. This metabolic shift expands nuclear NAD^+^ reserves and preserves SIRT1 activation, whereas inhibiting IDO1 reduces both nuclear NAD^+^ levels and SIRT1 function [78]. These findings may have direct implications for cardiometabolic diseases, which are characterized by chronic low-grade inflammation rather than the intense inflammatory burst seen in acute sepsis. In such conditions, the de novo pathway may be partially but not fully activated, leading to an intermediate state where upstream KYN metabolites accumulate while downstream flux to NAD^+^ remains inefficient. Such a dysregulated kynurenine pathway profile may underlie the paradoxical observation that elevated KYN and KTR coexist with impaired NAD^+^ homeostasis in disorders such as HFpEF [8,49].

Direct experimental evidence linking kynurenine pathway flux to myocardial NAD^+^ levels comes from a recent study by Li and colleagues using both animal and cell-culture models of doxorubicin (DOX)-induced cardiotoxicity [77]. The authors found that DOX treatment upregulates the enzyme α-amino-β-carboxy-muconate-semialdehyde decarboxylase (ACMSD) while downregulating QPRT, resulting in decreased QA and NAD^+^ concentrations [77]. These alterations were reversed by TES-1025, an ACMSD inhibitor that enhances QPRT activity and strengthens the cardioprotective capacity of the kynurenine pathway against DOX-induced cardiomyopathy [77]. Mechanistically, DOX was shown to operate through the STING/interferon-γ/AMP-activated protein kinase (AMPK) signaling axis to raise ACMSD levels and suppress QPRT [77]. This work provides direct evidence that impaired flux through the kynurenine-NAD^+^ axis, particularly at the QA to NAD^+^ conversion step, contributes to myocardial injury, and that restoring this flux confers cardioprotection [77]. As Verdin has emphasized, NAD^+^ plays a fundamental role in mitochondrial respiration and energy homeostasis, meaning that disruptions in kynurenine pathway flux can directly compromise cardiomyocyte energetics, especially under metabolically stressful conditions [89].

Beyond NAD^+^-related mechanisms, the accumulation of redox-active metabolites such as 3-HK may also impair cellular metabolism [90]. Using an activity-based metabolomics screen in human colon cancer cells, Buchanan and colleagues demonstrated that 3-HK disrupted tricarboxylic acid (TCA) cycle function, likely through impaired aconitase activity, while simultaneously depleting glutathione, increasing ROS, and inducing apoptosis [90]. Furthermore, KYNU knockdown increased intracellular 3-HK levels and was associated with enhanced ROS production and apoptotic signaling [90]. These findings raise the possibility that 3-HK accumulation in the heart, whether due to increased production or impaired KYNU-mediated metabolism, could similarly contribute to oxidative stress, mitochondrial dysfunction, and cardiomyocyte injury [90].

Importantly, emerging evidence indicates that KYN can also directly modulate cardiomyocyte signaling pathways. Mohiti et al. [91] reported that KYN induces apoptosis and autophagy in cardiomyocytes through activation of the AHR and downstream p38 mitogen-activated protein kinase (p38 MAPK) signaling. In this study, KYN increased apoptotic signaling and mitochondrial dysfunction, while inhibition of AHR or mitogen-activated protein kinase (MAPK) pathways attenuated these effects [91]. Resveratrol reduced apoptosis while promoting autophagy, suggesting that KYN-induced stress engages both pro-death and adaptive responses [91]. These findings provide mechanistic evidence that KYN can directly influence cardiomyocyte fate through AHR-dependent signaling [91].

However, not all kynurenine pathway metabolites exert deleterious effects. In contrast to KYN and 3-HK, KA has demonstrated cardioprotective properties. In a model of simulated ischemia–reperfusion injury, cardiomyocytes displayed pronounced apoptotic changes, including membrane blebbing, apoptotic nuclear morphology, DNA double-strand breaks, and caspase activation [56]. Notably, exogenous KA administration attenuated these morphological and molecular markers of apoptosis in a dose-dependent manner [56]. These protective effects occurred independently of N-methyl-D-aspartate (NMDA) receptor signaling, suggesting that NMDA receptor antagonism is unlikely to represent the principal mechanism underlying KA-mediated cardioprotection in this context [56].

Consistent with these findings, Olenchock et al. demonstrated that inhibition of the α-ketoglutarate (αKG)-dependent dioxygenase Egln1, an oxygen sensor involved in regulating hypoxia-inducible factors (HIF), protects against myocardial ischemia–reperfusion injury in mice [75]. This cardioprotective effect, observed following either systemic or skeletal muscle-specific Egln1 deletion, was mediated by a circulating factor. Mechanistically, Egln1 inhibition increased circulating αKG levels, which stimulated hepatic KA production; importantly, KA was shown to be both necessary and sufficient to confer cardiac protection [75].

In addition to direct effects, TRP metabolism may influence myocardial function indirectly through endothelial–cardiomyocyte crosstalk. Melhem et al. [5] reported that endothelial-specific modulation of IDO1 alters cardiac remodeling and function, and that KYN supplementation reverses these effects, supporting a role for endothelial-derived metabolites in regulating myocardial responses [5]. More broadly, integrative analyses, such as that of Wang et al., summarized that kynurenine pathway activation is closely linked to inflammatory signaling, oxidative stress, and metabolic dysregulation within cardiovascular tissues [92]. These integrated effects of TRP metabolism on endothelial function, vascular remodeling, and myocardial energetics reflect key pathophysiological processes underlying cardiometabolic diseases [1]. Accordingly, alterations in kynurenine pathway activity have been increasingly observed across conditions characterized by chronic inflammation and metabolic dysregulation [1], which are discussed in the following section.

## 4. Tryptophan Metabolism in Cardiometabolic Diseases

### 4.1. Obesity and Diabetes

**Figure 3:**
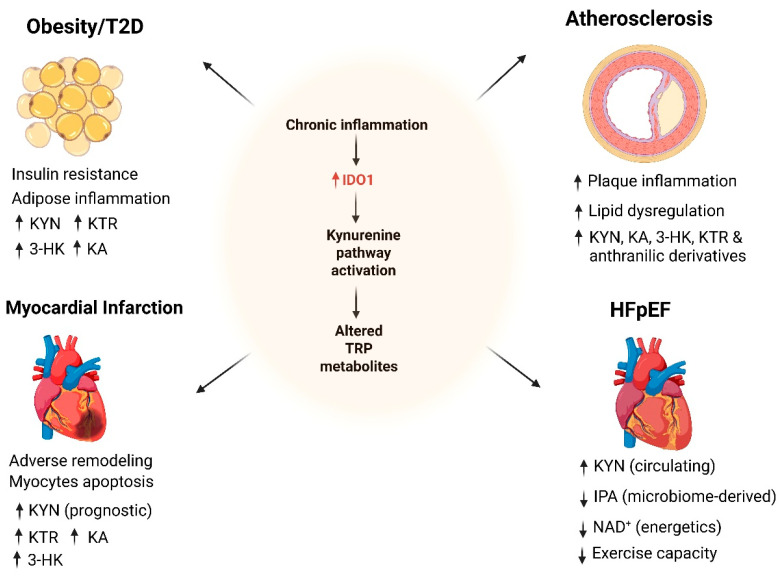
**Tryptophan (TRP) metabolism in cardiometabolic diseases.** Chronic inflammation induces indoleamine 2,3-dioxygenase 1 (IDO1), leading to activation of the kynurenine pathway and accumulation of altered TRP metabolites. In obesity and type 2 diabetes (T2D), kynurenine (KYN) pathway activation is associated with insulin resistance, adipose inflammation, and increased circulating KYN, kynurenine-to-tryptophan ratio (KTR), 3-hydroxykynurenine (3-HK), and kynurenic acid (KA). In atherosclerosis, elevated KYN metabolites are linked to plaque inflammation and lipid dysregulation. In myocardial infarction (MI), increased KYN pathway activity is associated with adverse remodeling, cardiomyocyte apoptosis, and prognostic elevation of circulating metabolites. In heart failure with preserved ejection fraction (HFpEF), kynurenine pathway activation and reduced microbiome-derived metabolites, such as indole-3-propionic acid (IPA), are associated with impaired energetics, reduced nicotinamide adenine dinucleotide (NAD^+^) availability, and decreased exercise capacity. ↑ and ↓ indicate increased and decreased levels, ratios, expression/activity, or disease related features, respectively.

Obesity, metabolic syndrome, and type 2 diabetes (T2D) are characterized by chronic low-grade inflammation and metabolic alterations that have been associated with changes in TRP metabolism along the kynurenine pathway [9,10,12,13,16,17]. Several studies indicate that inflammatory signaling can enhance the activity of IDO1, thereby promoting the conversion of TRP into KYN [13]. As summarized by Kozieł and Urbanska [13], pro-inflammatory mediators such as IFN-γ, TNF-α, and LPS can induce IDO1 activity, suggesting a link between inflammation and kynurenine pathway activation (Figure 3) [9]. In clinical settings, alterations in kynurenine pathway metabolites have been reported in individuals with obesity and metabolic dysfunction. Favennec et al. [11] observed that circulating levels of KYN, KA, and QA were higher in individuals with obesity and were positively associated with body mass index (BMI) and insulin resistance indices [11]. In the same study, the expression of several kynurenine pathway enzymes, including IDO1, KYNU, and KMO, was increased in omental adipose tissue, and their expression was inducible by pro-inflammatory cytokines in vitro, suggesting that adipose tissue may contribute to kynurenine pathway alterations [11].

Experimental data provide additional insights into potential mechanisms [12]. Huang et al. [12] reported that obesity increases IDO1 expression in mature adipocytes, enhancing TRP catabolism and KYN production in white adipose tissue and circulation, while stromal vascular fraction and macrophage-specific knockout data suggested that macrophage IDO1 was not the critical driver [12]. KYN activates the AHR, which binds the signal transducer and activator of transcription 3 (STAT3) promoter, increasing STAT3 expression and phosphorylation and promoting IL-6 production [12]. This IDO1–KYN–AHR–STAT3–IL-6 axis increased CCAAT/enhancer-binding protein alpha (C/EBPα), peroxisome proliferator-activated receptor gamma (PPARγ), and fatty acid-binding protein 4 (FABP4), reduced phosphorylation of hormone-sensitive lipase at Ser660 (p-HSL^Ser660^), decreased inhibitory phosphorylation of acetyl-CoA carboxylase at Ser79 (p-ACC^Ser79^), and impaired insulin signaling through reduced phosphorylation of protein kinase B (AKT) at Ser473 (p-AKT^Ser473^), thereby promoting lipogenesis, suppressing lipolysis, and contributing to insulin resistance [12]. IFN-γ further induced adipocyte IDO1 and downstream AHR/STAT3/IL-6 signaling, whereas AHR inhibition with StemRegenin 1, phosphorylated STAT3 inhibition with Stattic, IL-6 receptor blockade with Tocilizumab, adipocyte-specific deletion of IDO1 or AHR, and vitamin B6/PLP-facilitated KYN catabolism attenuated this pathway [12]. These findings suggest that adipocyte-derived KYN may connect kynurenine pathway activation with inflammatory and metabolic dysfunction in obesity, although its precise contribution in humans remains to be fully established.

Complementary experimental evidence suggests that IDO1 may also regulate obesity-associated metabolic dysfunction through intestinal TRP metabolism and gut microbiota-dependent mechanisms. Laurans et al. [15] showed that genetic IDO1 deletion protected mice from high-fat diet-induced body weight gain, adiposity, liver steatosis, adipose inflammation, endotoxemia, and insulin resistance, whereas pharmacological inhibition with L-1-methyl-tryptophan (1 MT) improved insulin sensitivity without significantly reducing body weight [15]. This protection was not explained simply by reduced KYN, since KYN supplementation did not alter body weight, white adipose tissue weight, or insulin sensitivity, and KA supplementation did not alter body weight in Ido1−/− mice [15]. Instead, IDO1 deficiency shifted intestinal TRP metabolism away from KYN production toward microbiota-dependent indole derivatives, particularly IAA, enhancing AHR activation, Interleukin-22 (IL-22) and Interleukin-17 (IL-17) production in Peyer’s patches, and IL-22 target genes regenerating islet-derived protein 3 beta (Reg3b) and regenerating islet-derived protein 3 gamma (Reg3g), thereby preserving gut barrier function and reducing circulating LPS [15]. Antibiotic depletion, co-housing, fecal microbiota transfer, and IL-22 neutralization experiments further supported a microbiota- and IL-22-dependent mechanism [15]. Together with the adipocyte-focused findings above, these data suggest that IDO1 may contribute to obesity-associated metabolic dysfunction through both tissue-intrinsic KYN signaling and gut microbiota-dependent diversion of TRP away from indole–AHR–IL-22 pathways that support intestinal barrier integrity and metabolic homeostasis [15].

In contrast, certain downstream kynurenine pathway metabolites may exert metabolically favorable effects. Wang et al. [18] identified KA as an endogenous ligand for GPR35, showing that KA selectively activated human, mouse, and rat GPR35, induced intracellular calcium signaling, inositol phosphate accumulation, pertussis toxin-sensitive guanosine 5′-O-(3-thiotriphosphate) (GTPγS) binding, and GPR35 internalization [18]. GPR35 was predominantly expressed in immune and gastrointestinal tissues, and KA attenuated LPS-induced TNF-α secretion in PBMCs and cluster of differentiation 14-positive (CD14+) monocytes, suggesting a potential immunomodulatory role [18]. Agudelo et al. [76] extended these findings to metabolic regulation, showing that KA activates GPR35 in adipose tissue, increases energy expenditure, reduces adiposity, improves glucose tolerance, and induces thermogenic, lipid oxidative, and anti-inflammatory gene programs [76]. Mechanistically, KA–GPR35 signaling increased calcium release, extracellular signal-regulated kinase (ERK), and CREB signaling, stabilized peroxisome proliferator-activated receptor gamma coactivator 1-alpha 1 (PGC-1α1), increased regulator of G protein signaling 14 (RGS14), and enhanced beta-adrenergic receptor (β-AR) signaling in adipocytes [76]. Conversely, GPR35 deletion promoted weight gain and glucose intolerance, increased susceptibility to high-fat diet-induced metabolic dysfunction, and impaired exercise-induced adipose tissue browning [76]. These studies suggest that KA–GPR35 signaling may counter metabolic dysfunction by promoting adipose energy expenditure and anti-inflammatory responses; however, within obesity, the effects of kynurenine pathway activation may differ according to the specific metabolite, receptor pathway, and tissue compartment involved, such that potentially beneficial KA–GPR35 signaling should be distinguished from upstream KYN accumulation and adipocyte KYN–AHR–STAT3–IL-6 signaling [18,76].

Alterations in kynurenine pathway metabolites have also been described in individuals with T2D. Kubacka et al. [14] reported that obese women with T2D had higher circulating TRP and KA, as well as increased KA/QA and KA/3-HK ratios compared to normoglycemic obese women. TRP, KA, and particularly the KA/3-HK ratio were most strongly associated with T2D in logistic regression analysis, with a model including TRP and this ratio explaining approximately 20% of the variability in disease presence. In addition, the relationships between KYN metabolites and metabolic parameters differed between groups, with stronger associations between BMI and kynurenine pathway metabolites observed in normoglycemic obese women. Collectively, increased TRP and KA levels, together with a higher KA/3-HK ratio, were associated with the occurrence of T2D in this population [14]. More broadly, Kozieł and Urbańska [13] summarized evidence from human and experimental studies indicating that inflammatory mediators such as IFN-γ, TNF-α, and LPS can induce IDO1 activity, thereby enhancing kynurenine pathway flux [13]. The authors further describe that KYN metabolites exert diverse and sometimes opposing biological effects, including pro- and anti-inflammatory, as well as oxidant and antioxidant actions, depending on their concentration and cellular context [13]. Alterations in kynurenine pathway metabolites and the KYN/TRP ratio were reported across studies in diabetes and metabolic disorders, supporting an association between kynurenine pathway dysregulation and metabolic disease, although causal relationships remain to be established [13]. Additional studies have reported associations between kynurenine pathway metabolites, adiposity, and systemic inflammatory markers across different populations [9,16,17].

Overall, current evidence supports an association between kynurenine pathway activation and metabolic conditions, including obesity and T2D. Inflammatory signaling appears to promote IDO1 activity and TRP conversion into KYN, while experimental studies indicate that kynurenine pathway activation may affect metabolic regulation through multiple mechanisms, including adipocyte KYN–AHR–STAT3–IL-6 signaling, gut microbiota-dependent regulation of intestinal indole–AHR–IL-22 pathways, and KA–GPR35-mediated effects on adipose energy expenditure and inflammation. However, causal relationships in humans remain incompletely established, and further studies are required to clarify the relative contribution of individual metabolites, receptor pathways, and tissue compartments in obesity and diabetes.

### 4.2. Atherosclerosis

Atherosclerosis is a chronic inflammatory disease of the arterial wall driven by the interplay between lipid accumulation and immune activation [1]. Both innate and adaptive immune responses contribute to disease progression, with macrophages, dendritic cells, and T- and B-lymphocytes orchestrating inflammatory signaling in response to modified lipoproteins such as oxLDL [1,2]. This leads to the production of pro-inflammatory mediators, including IL-1β, IL-6, and TNF-α, as well as an imbalance between regulatory and effector immune responses that promotes plaque progression and instability [1,2]. Within this inflammatory environment, activation of TRP metabolism, particularly through the kynurenine pathway, has been consistently observed [1,2]. Clinical studies indicate that increased kynurenine pathway activity, reflected by elevated KTR and higher levels of metabolites such as KYN, KA, 3-HK, and anthranilic derivatives, is associated with atherosclerotic burden and cardiovascular risk (Figure 3) [2]. In patients with advanced atherosclerosis, higher KTR correlates with plaque presence, postoperative complications, and adverse cardiovascular outcomes [21]. Moreover, Baumgartner et al. [19] reported a deviation of TRP metabolism within the kynurenine pathway in human carotid atherosclerotic disease. Transcriptomic analysis of carotid plaques and control arteries showed increased expression of enzymes involved in the first step of TRP catabolism and the quinolinic branch, including tryptophan-2,3-dioxygenase (TDO2), IDO1, indoleamine 2,3-dioxygenase 2 (IDO2), KMO, and KYNU, together with reduced expression of the KA-branch enzymes kynurenine aminotransferase 1 (KAT1) and kynurenine aminotransferase 2 (KAT2) [19]. Analysis of plaques from patients with cerebrovascular symptomatic and asymptomatic carotid stenosis further showed increased KMO and KYNU expression, reduced KAT2 transcript levels, lower KAT1 protein expression, and a reduced local KA/3-HAA ratio in symptomatic patients compared with asymptomatic patients, indicating impaired KA-branch activity [19]. In vitro, KA reduced TNF-α and IL-6 secretion, as well as CCR7, CCL5, and CXCL10 expression, in activated human monocyte-derived macrophages. Pharmacological experiments using AHR and GPR35 agonists and antagonists indicated that these anti-inflammatory effects were mediated mainly through AHR rather than GPR35 signaling [19]. In vivo, KA reduced Ly6G+ leukocyte recruitment and accelerated inflammatory resolution in a zymosan-induced mouse model of peritoneal inflammation [19].

Experimental studies suggest that the functional role of the kynurenine pathway in atherosclerosis is complex and context-dependent. IDO1 has been implicated in both protective and pro-inflammatory mechanisms [1,2]. In apolipoprotein E-deficient (ApoE−/−) mice, genetic IDO deficiency increased lesion size and features of plaque vulnerability, including increased lesional macrophage and T-cell accumulation, reduced smooth muscle cell (SMC)-positive plaque area, and increased necrotic core area [23]. This was associated with reduced circulating KYN without altered TRP levels, reduced serum interleukin-10 (IL-10), and fewer IL-10-producing B cells, suggesting that loss of downstream TRP metabolites may impair anti-inflammatory B-cell responses during atherogenesis [23]. Consistently, 3,4-dimethoxycinnamoyl anthranilic acid (3,4-DAA), a synthetic anthranilic acid derivative, increased IL-10-producing B cells in vitro, reduced lesion formation and macrophage recruitment after arterial injury in ApoE−/− mice, and decreased inflammatory mediator production in ex vivo human atheroma cultures [23]. In a complementary model, IDO inhibition with 1 MT in high-fat diet-fed ApoE−/− mice increased lesion size, vascular cell adhesion molecule-1 (VCAM-1), C-C motif chemokine ligand 2 (CCL2), TNF-α, CD^68+^ macrophage accumulation, and medial SMC VCAM-1 expression [26]. In human SMCs, 1-MT increased IFN-γ-induced VCAM-1 mRNA and protein, whereas 3-HAA reversed this effect; 3-HAA also halted 1-MT-induced lesion progression and reduced VCAM-1/CD68 responses in vivo [26]. Together, these studies suggest that IDO-derived metabolites restrain atherosclerosis by supporting IL-10-producing B-cell responses and limiting vascular adhesion/chemokine signaling, macrophage recruitment, and SMC inflammatory activation [23,26].

At the cellular level, IDO1 activity appears to regulate vascular immune tolerance through coordinated regulatory T cell–dendritic cell (Treg–DC) and plasmacytoid dendritic cell–regulatory T cell (pDC–Treg) pathways. Forteza et al. [24] showed that activation of the Treg/IDO1 axis using apolipoprotein B100 (ApoB100)-pulsed, transforming growth factor beta 2 (TGFβ2)-treated tolerogenic dendritic cells (DCs) expanded forkhead box P3(FoxP3) + regulatory T cells (Tregs), increased vascular IDO1 expression and local KYN staining, and reduced atherosclerotic lesion area, CD^68+^ macrophage infiltration, and VCAM-1 expression [24]. Mechanistically, cytotoxic T-lymphocyte-associated protein 4 (CTLA4), a key Treg effector molecule, enhanced IFN-γ-induced IDO1 expression and KYN/TRP activity in human smooth muscle cells, endothelial cells, and macrophages, supporting a vascular tolerance loop in which Tregs promote IDO1-dependent TRP metabolism and reinforce anti-inflammatory immune regulation [24]. Yun et al. [28] further identified IDO1-expressing aortic plasmacytoid dendritic cells (pDCs) as an atheroprotective population that promotes local Treg induction; selective pDC depletion aggravated atherosclerosis and reduced aortic Tregs and IL-10, while pDCs and Tregs colocalized in the atherosclerotic intima [28]. Together, these studies support a protective IDO1–KYN–Treg tolerance axis in the vessel wall [24,28]. However, IDO1 signaling may also contribute to plaque thrombogenicity under inflammatory macrophage activation. Watanabe et al. [27] showed that IDO1 was mainly localized in CD^68+^ macrophage-rich regions of human coronary plaques and was closely associated with tissue factor (TF), with larger IDO1, TF, and CD3-positive areas in unstable than stable angina plaques [27]. In activated macrophages, IFN-γ and TNF-α increased IDO1 expression, KYN/TRP ratio, and TF expression/activity, whereas IDO1 inhibition with epacadostat reduced the KYN/TRP ratio, TF expression/activity, and NF-κB p65 binding activity [27]. In addition, KYN increased TF expression and AHR nuclear translocation, while AHR inhibition reduced KYN-induced TF expression [27]. These findings suggest an IDO1–KYN–AHR/NF-κB–TF pathway that may link inflammatory macrophage activation to plaque thrombogenicity, although the in vivo contribution of this pathway to atherothrombosis requires further studies [27].

Downstream kynurenine pathway metabolites further contribute to these divergent effects. 3-HAA, for example, has been shown to reduce atherosclerotic lesion size, attenuate vascular inflammation, and modulate lipid metabolism in experimental models [20,26]. Mechanistically, Berg et al. [20] showed that 3-HAA acts through both inflammatory and lipid-metabolic pathways [20]. In macrophages, 3-HAA inhibited inflammasome activation by reducing cleaved caspase-1 and IL-1β secretion after LPS priming and adenosine triphosphate (ATP) stimulation, while in hepatocyte models, it suppressed sterol regulatory element-binding protein 2 (SREBP-2) expression and nuclear translocation and reduced apolipoprotein B (ApoB) secretion [20]. In LDL receptor-deficient (Ldlr−/−) mice, inhibition of 3-HAA 3,4-dioxygenase (HAAO), which increases endogenous 3-HAA, reduced plasma cholesterol and triglycerides, decreased atherosclerotic lesion area, lowered hepatic SREBP-2 and 3-hydroxy-3-methylglutaryl-coenzyme A (HMG-CoA) reductase expression, and improved liver lipid accumulation and inflammation [20]. In addition, 3-HAA may influence early foam-cell formation by limiting both LDL oxidative modification and macrophage uptake of oxidized LDL [22,29]. In PBMCs and monocyte-derived macrophages, IFN-γ-induced TRP metabolism generated 3-HAA, which contributed to the attenuation of cell-mediated LDL oxidation, while exogenous 3-HAA directly inhibited LDL oxidation [22]. In macrophages from 3-HAA-treated Ldlr−/− mice and in RAW264 macrophages, 3-HAA reduced oxLDL uptake, suggesting an additional mechanism by which this metabolite may limit macrophage lipid loading during early plaque formation [29]. However, many of these effects have been observed under experimental conditions, and their physiological relevance remains to be fully established. In parallel, enzyme-level regulation within the kynurenine pathway appears to influence disease phenotype. Together with the Baumgartner et al. [19] findings described above, these data suggest that vascular outcomes may depend on the balance between KMO/KYNU-driven quinolinic-branch activity and KAT-mediated KA production, rather than overall kynurenine pathway activation alone [19].

Beyond the kynurenine pathway, alternative TRP metabolic pathways may also contribute to atherosclerosis [1]. Serotonin signaling has been implicated in vascular inflammation, endothelial permeability, and leukocyte adhesion, while melatonin has been shown to improve endothelial function and reduce plaque instability in preclinical models [1]. These observations highlight that TRP metabolism operates as an interconnected network influencing vascular biology through multiple pathways. Taken together, current evidence indicates that TRP metabolism contributes to atherosclerosis through integrated effects on immune regulation, lipid metabolism, and vascular inflammation (Figure 3). However, the overall impact of the kynurenine pathway appears to be highly context-dependent, influenced by disease stage, metabolic conditions, and the relative balance of downstream metabolites. A more precise understanding will require studies that address cell-specific pathway activity and distinguish between the effects of individual metabolites within the TRP metabolic network.

### 4.3. Myocardial Infarction

MI results from coronary artery occlusion following plaque rupture or erosion, leading to myocardial ischemia and necrosis. Despite advances in revascularization, adverse left ventricular (LV) remodeling and HF remain major determinants of long-term outcomes [1,40]. These processes are largely driven by inflammatory responses, highlighting the relevance of metabolic pathways that modulate post-ischemic remodeling [1]. Activation of the kynurenine pathway has been consistently associated with cardiovascular risk and MI. Prospective studies (e.g., Sulo et al. [38]; Pedersen et al. [35]) show that elevated KTR predicts future coronary events, including MI [35,38]. Similarly, higher plasma KTR is associated with major coronary events, cardiovascular mortality, and all-cause mortality [41]. In patients with stable angina, increased levels of KYN metabolites, including KA, 3-HK, and anthranilic derivatives, are linked to higher MI risk, particularly in the presence of metabolic dysfunction (Figure 3) [36].

Kynurenine pathway activation also reflects myocardial injury severity and remodeling. Experimental studies report increased circulating KYN metabolites after ischemic injury, with higher KA and 3-HAA acid levels correlating with reduced ejection fraction and impaired cardiac function [31,37]. Clinically, increased IDO1 activity is associated with adverse LV remodeling after MI and may outperform established biomarkers such as N-terminal pro-B-type natriuretic peptide (NT-proBNP), suppression of tumorigenicity 2 (ST2), and C-reactive protein (CRP) in predicting remodeling outcomes [39]. Mechanistic studies indicate that IDO1-driven KYN production contributes to post-MI remodeling through endothelial–cardiomyocyte interactions [5]. Melhem et al. showed that endothelial-specific IDO1 deletion or inhibition improves cardiac function and attenuates remodeling, whereas KYN reverses these effects via AHR-dependent ROS generation and cardiomyocyte apoptosis [5]. Consistent with this, ischemia-induced IDO1 activation appears to be driven by inflammatory signaling and primarily involves non-hematopoietic cells, particularly endothelial cells [1,5]. However, kynurenine pathway signaling is not uniformly deleterious. KYN has been reported to mediate protective effects of remote ischemic preconditioning, and its administration prior to ischemia reduces infarct size in experimental models [30,32]. These findings underscore the context-dependent nature of kynurenine pathway activation. Beyond the kynurenine pathway, other TRP pathways contribute to MI pathophysiology. Serotonin promotes neutrophil activation, leukocyte adhesion, and inflammatory injury during ischemia–reperfusion, whereas inhibition of serotonin signaling reduces inflammation and preserves cardiac function [34]. In contrast, melatonin exerts cardioprotective effects by limiting oxidative stress, apoptosis, and mitochondrial dysfunction, with experimental and clinical studies supporting its role in improving post-MI outcomes [33]. Overall, TRP metabolism plays a multifaceted and context-dependent role in MI, influencing both injury and repair through effects on inflammation, oxidative stress, and cellular signaling. While kynurenine pathway activation is consistently associated with adverse outcomes, further studies are required to define the specific contributions of individual metabolites and their therapeutic potential in MI.

### 4.4. Heart Failure with Preserved Ejection Fraction

Alterations in TRP metabolism are increasingly recognized as a biologically relevant feature of HFpEF [42,43,44,45,46,47,48,49,50,51,52]. Although direct mechanistic interrogation of TRP-derived pathways in HFpEF has historically been limited, recent human metabolomic studies, together with emerging experimental evidence, now link disrupted TRP metabolism to impaired myocardial energetics, inflammation, and vascular dysfunction in this syndrome [8,42,43,44,45,46,47,48,49,50,51,52]. The most direct HFpEF-specific evidence implicating altered TRP metabolism in disease pathophysiology was provided by Wang et al. [8], who performed comprehensive analyses in two independent human HFpEF cohorts combined with mechanistic studies in a validated murine model of HFpEF [8]. In this study, circulating levels of the microbiome-derived TRP metabolite IPA were significantly reduced in patients with HFpEF, whereas both plasma and myocardial IPA levels were decreased in a mouse model of HFpEF (Figure 3) [8]. Importantly, lower IPA levels were associated with greater diastolic dysfunction and metabolic impairment, linking disruption of a specific TRP-derived pathway to clinically relevant HFpEF phenotypes (Figure 3) [8].

Using a “two-hit” murine HFpEF model, Wang et al. demonstrated that dietary supplementation with IPA attenuated left ventricular hypertrophy, myocardial fibrosis, inflammation, and diastolic dysfunction while preserving ejection fraction. Mechanistically, IPA restored nicotinamide and NAD^+^ availability through suppression of nicotinamide N-methyltransferase (NNMT) and activation of the AHR– Sirtuin 3 (SIRT3) signaling axis. Cardiac-specific knockdown of SIRT3 abolished the beneficial effects of IPA, establishing a causal link between altered TRP metabolism, impaired mitochondrial energetics, and HFpEF pathophysiology [8]. Together, these findings provide HFpEF-specific evidence supporting a mechanistic link between TRP metabolism and disease pathophysiology [8]. In parallel with these findings, activation of the KYN branch of TRP metabolism has been consistently reported in HFpEF. Using targeted LC–MS/MS metabolomic profiling, Lewkowicz et al. [49] showed that patients with HFpEF, particularly those with T2D, exhibited increased circulating concentrations of TRP, KYN, and anthranilic acid (AA), accompanied by relatively reduced levels of downstream metabolites such as 3-HK and QA. These alterations were associated with adverse cardiac remodeling, including left ventricular hypertrophy, left atrial enlargement, and impaired global longitudinal strain, suggesting a link between kynurenine pathway dysregulation and subclinical myocardial dysfunction [49]. Consistent with these observations, in patients with new-onset HF, Hage et al. reported that HFpEF was associated with cardiometabolic comorbidities and a distinct metabolic profile characterized by increased inflammation and oxidative stress, impaired NO signaling, and enhanced collagen synthesis [45]. Circulating KYN levels were higher in HFpEF, particularly in patients with diabetes and kidney dysfunction [45]. Extending these metabolic observations to functional phenotypes, Bekfani et al. [43] demonstrated that circulating KYN concentrations were significantly elevated in HFpEF patients with reduced skeletal muscle endurance and were independently associated with impaired quadriceps endurance and shorter six-minute walk distance, with good discriminatory performance for reduced muscle endurance (AUC 0.83) [43]. Collectively, these studies suggest that kynurenine pathway activation in HFpEF is associated with functional impairment [93].

Broader metabolomic analyses employing targeted pathway enrichment and multivariate modeling further reinforce the prominence of TRP metabolism in HFpEF [47,48]. Kozhevnikova et al. [48] demonstrated that multiple TRP-derived metabolite, including 3-HK, AA, TRP, and the KTR, contributes prominently to metabolic signatures distinguishing HFpEF from hypertension and HFrEF. In a complementary study applying the same targeted analytical platform across heart-failure stages and ejection-fraction phenotypes, TRP-derived metabolites again ranked among the most informative features for heart-failure phenotyping and were associated with disease severity and adverse outcomes [47]. Additional untargeted metabolomic studies confirmed that TRP metabolism is among the most consistently dysregulated pathways in HFpEF [42,44]. Experimental tissue-level analyses using vibrational spectroscopy have further demonstrated reduced myocardial TRP content and abnormal extracellular-matrix chemistry in HFpEF, providing important insight into how altered TRP metabolism may translate into myocardial structural and biochemical remodeling [51]. Collectively, these HFpEF-specific metabolomic studies indicate that activation of the kynurenine pathway is a robust and reproducible metabolic feature of the syndrome, particularly in patients with cardiometabolic comorbidities. However, the observed accumulation of upstream metabolites alongside relative depletion of downstream NAD^+^-producing intermediates suggests inefficient metabolic resolution toward adaptive energetics, consistent with chronic inflammatory activation rather than beneficial remodeling [42,44,45,47,48,49].

Importantly, these HFpEF-specific metabolomic signatures align with observations from broader HF and cardiometabolic populations, in which kynurenine pathway activation has been consistently linked to impaired functional capacity and adverse clinical outcomes [46]. In the SICA-HF cohort, Konishi et al. [46] demonstrated that plasma KYN concentrations, measured using a targeted approach, were elevated in both HFpEF and HFrEF compared with controls and were independently associated with reduced peak oxygen consumption, lower muscle strength, and increased mortality, without corresponding reductions in skeletal muscle mass, underscoring a functional rather than structural phenotype [46]. Extending these functional observations to long-term prognosis, Lund et al. [50] using targeted LC–MS/MS quantification of a comprehensive kynurenine pathway panel, showed that higher circulating levels of KYN, 3-HK, QA, the KTR, and the 3-HK/ xanthurenic acid (XA) ratio were independently associated with increased mortality in patients with established HF, whereas XA was inversely associated with risk, highlighting marked metabolite-specific heterogeneity within the pathway [50]. In individuals without baseline HF, higher baseline levels of KTR, KA, and QA, measured by targeted LC–MS/MS, were found to predict the incident of HF [52]. Although QA was associated with atrial fibrillation, these associations were absent in individuals randomized to a Mediterranean diet, suggesting that inflammatory activation of TRP metabolism precedes clinical HF and may be modifiable [52].

Altogether, these HFpEF-specific human and experimental studies indicate that disruption of TRP metabolism encompassing both microbiome-derived indole pathways and activation of the KYN branch is a consistent metabolic feature of the syndrome and is associated with adverse myocardial structure, impaired energetics, reduced exercise capacity, and worse clinical outcomes (Figure 3) [8,42,43,44,45,46,47,48,49,50,51]. However, the majority of available HFpEF data are derived from circulating metabolomic analyses or whole-tissue measurements and therefore cannot define the cellular sources of altered TRP metabolism. Taken together, these findings identify TRP metabolism as a relevant metabolic axis in HFpEF, linking inflammatory activation with alterations in myocardial energetics and functional capacity. However, further studies are required to define the cellular sources and causal contribution of specific TRP-derived metabolites in this syndrome.

## 5. Therapeutic Opportunities and Future Perspectives

### 5.1. The Kynurenine-to-Tryptophan Ratio and Downstream Metabolite Panels as Clinical Biomarkers

The KTR is the most widely used clinical surrogate of kynurenine pathway activation. Because both IDO1 and TDO catalyze the initial conversion of TRP toward KYN, an increased KTR indicates enhanced TRP catabolism, but it does not identify the exact cellular source or enzyme responsible. In cardiometabolic disease, elevated KTR has been associated with coronary events, MI risk, cardiovascular mortality, HF outcomes, and impaired functional capacity, supporting its potential utility as a risk marker across inflammatory and metabolic cardiovascular phenotypes [35,38,41,50,52]. However, KTR should be interpreted as an entry-point biomarker rather than a disease-specific diagnostic marker. It reflects activation of a shared inflammatory–metabolic pathway that may occur across atherosclerosis, MI, HF, obesity, and T2D. Therefore, its clinical value depends on the setting in which it is measured. In patients with established atherosclerotic disease, elevated KTR may reflect heightened inflammatory pathway activation and increased risk of adverse cardiovascular outcomes, whereas in HF, it may reflect systemic inflammation, cardiometabolic comorbidity burden, kidney dysfunction, impaired functional capacity, or adverse prognosis [21,41,46,50,52]. Thus, KTR may be useful for risk stratification or monitoring pathway activation within defined patient groups, but not for distinguishing one cardiometabolic disease from another.

**Figure 4:**
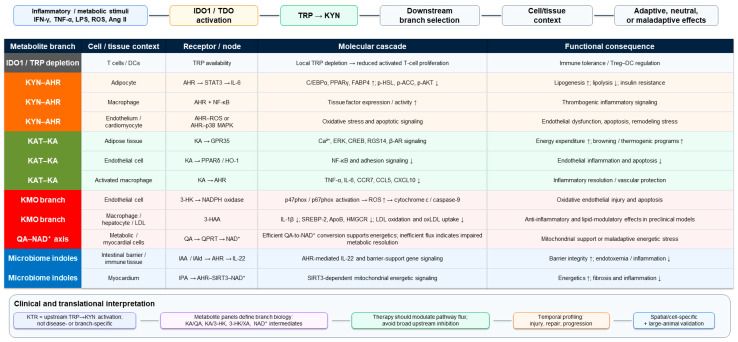
**Branch and cell-specific mechanisms downstream of tryptophan metabolism in cardiometabolic diseases.** Inflammatory and metabolic stimuli, including interferon-γ (IFN-γ), tumor necrosis factor-α (TNF-α), lipopolysaccharide (LPS), reactive oxygen species (ROS), and angiotensin II (Ang II), induce indoleamine 2,3-dioxygenase 1 (IDO1) and/or tryptophan-2,3-dioxygenase (TDO), promoting tryptophan (TRP) conversion into kynurenine (KYN). The biological consequences of this activation depend on the downstream branch engaged, the cell or tissue compartment involved, and the signaling node activated. IDO1-mediated local TRP depletion can limit activated T-cell proliferation and support regulatory T-cell/dendritic-cell (Treg/DC)-mediated immune tolerance. KYN can activate aryl hydrocarbon receptor (AHR)-dependent signaling in adipocytes, macrophages, endothelial cells, and cardiomyocytes, linking TRP metabolism to impaired insulin signaling, tissue factor expression, oxidative stress, apoptosis, and remodeling. In contrast, conversion of KYN through kynurenine aminotransferases (KATs) to kynurenic acid (KA) may support regulatory or cytoprotective signaling through G protein-coupled receptor 35 (GPR35), peroxisome proliferator-activated receptor delta (PPARδ)/heme oxygenase-1 (HO-1), or AHR, depending on tissue context. Flux through the kynurenine 3-monooxygenase (KMO) branch generates 3-hydroxykynurenine (3-HK), 3-hydroxyanthranilic acid (3-HAA), and quinolinic acid (QA), with divergent effects on redox signaling, lipid metabolism, inflammation, and nicotinamide adenine dinucleotide (NAD^+^)-linked metabolic resolution. In parallel, microbiome-derived indole metabolites, including indole-3-acetic acid (IAA), indole-3-aldehyde (IAld), and indole-3-propionic acid (IPA), regulate gut barrier integrity, inflammation, and myocardial energetics through AHR-, interleukin-22 (IL-22)-, sirtuin 3 (SIRT3)-, and NAD^+^-linked pathways. The lower panel summarizes translational implications: the kynurenine-to-tryptophan ratio (KTR) reflects upstream TRP-to-KYN activation, whereas downstream metabolite panels, temporal profiling, spatial/cell-specific analyses, and large-animal validation are needed to define branch-specific biology and guide pathway-informed therapeutic strategies. ↑ and ↓ indicate upregulation/increase and downregulation/decrease of the indicated molecular signal, pathway activity, or functional outcome, respectively.

A key limitation of KTR is that it does not define downstream pathway routing. This distinction is clinically important because the biological consequences of kynurenine pathway activation depend on whether KYN is directed toward KA-associated regulatory signaling, KMO/3-HK/QA-associated redox activity, 3-HAA-linked lipid and inflammasome regulation, microbiome-derived indole pathways, or NAD^+^-generating metabolic resolution. Accordingly, metabolite panels combining KTR with KYN, KA, 3-HK, 3-HAA, QA, XA, KA/QA, KA/3-HK, 3-HK/XA, and NAD^+^-related intermediates may provide more clinically meaningful information than KTR alone [14,19,49,50,52]. Such panels may help distinguish regulatory or compensatory profiles from patterns dominated by oxidative stress, inflammatory activation, or inefficient NAD^+^ metabolic resolution (Figure 4).

### 5.2. Therapeutic Targeting of TRP Metabolism

Therapeutic targeting of TRP metabolism is promising but challenging because the kynurenine pathway contains both protective and detrimental signaling axes. Broad upstream inhibition of IDO1 or TDO is therefore unlikely to be uniformly beneficial in cardiometabolic diseases [3,4,6,18,19,55,56,68,69,75,76]. Although excessive KYN–AHR signaling may contribute to inflammatory or stress responses in specific cellular contexts, IDO1-derived metabolites can also support immune tolerance, vascular protection, and NAD^+^ metabolism [15,23,24,26]. In atherosclerosis models, genetic IDO deficiency or pharmacological IDO inhibition aggravated lesion formation and inflammatory vascular responses, whereas downstream metabolites such as 3-HAA or anthranilic acid derivatives attenuated vascular inflammation [23,26]. Therefore, future cardiometabolic strategies may need to modulate downstream branch activity or restore beneficial metabolite signaling rather than broadly suppressing upstream TRP catabolism (Table 1) [19].

Upstream enzyme inhibition has been developed most extensively in oncology. IDO1 inhibitors such as epacadostat and navoximod, as well as dual IDO1/TDO inhibitors such as M4112, have entered clinical testing mainly in advanced solid tumors [94]. Epacadostat was evaluated in the phase III ECHO-301/KEYNOTE-252 melanoma trial but did not improve progression-free or overall survival when added to pembrolizumab, highlighting the limitations of pathway inhibition without adequate pharmacodynamic monitoring or pathway-defined patient selection [95]. Navoximod has been evaluated in phase I testing in recurrent or advanced solid tumors, and M4112 has been reported as an oral dual IDO1/TDO2 inhibitor in first-in-human oncology testing [96,97]. At present, these agents have not established a therapeutic role in cardiometabolic diseases. Their potential translation to cardiometabolic indications would require careful attention to disease context, timing, tissue-specific pathway activity, and downstream metabolite profiles [23,24,26].

More selective approaches may be better suited to cardiometabolic disease. In phenotypes dominated by excessive KMO/3-HK/QA-branch activity, strategies that reduce redox-active metabolite accumulation or redirect KYN away from 3-HK generation may help limit oxidative stress. If the dominant abnormality is impaired QA-to-NAD^+^ conversion, as suggested in DOX-induced cardiotoxicity models, restoring downstream NAD^+^ flux may be more appropriate than inhibiting upstream IDO1 [77]. Conversely, if protective metabolites are reduced or functionally insufficient, approaches that enhance KA, 3-HAA, or microbiome-derived indoles may be more relevant. KA-related strategies may support endothelial protection, adipose energy expenditure, macrophage anti-inflammatory signaling, or cardioprotection, depending on receptor and tissue context [18,19,55,56,75,76]. Similarly, 3-HAA-related approaches may reduce macrophage inflammasome activation, hepatic lipid synthesis, LDL oxidation, and macrophage oxLDL uptake in experimental systems [20,22,29].

Microbiome-directed interventions represent another therapeutic opportunity. In obesity-related metabolic dysfunction, IDO1 deficiency shifted intestinal TRP metabolism toward microbiota-derived indole metabolites and improved gut barrier and metabolic phenotypes through AHR–IL-22-dependent mechanisms [15]. In HFpEF, supplementation with the microbiome-derived indole metabolite IPA improved myocardial NAD^+^ availability, mitochondrial signaling, fibrosis, inflammation, and diastolic function in an experimental model [8]. These findings suggest that dietary, prebiotic, probiotic, postbiotic, or metabolite-based strategies could be explored to restore beneficial TRP-derived indole signaling, although clinical translation will require careful attention to microbiome variability, dose, bioavailability, safety, and disease stage.

### 5.3. Clinical Trials and Translational Opportunities

Most clinical trials targeting TRP or kynurenine pathway metabolism have been conducted outside cardiometabolic diseases, particularly in oncology, sleep, psychiatry, and metabolic-response studies. Nevertheless, these studies show that TRP availability and kynurenine pathway enzymes can be manipulated in humans. ClinicalTrials.gov includes NCT04505800, which evaluated oral TRP supplementation in night-shift workers, and NCT02184832, which examined dietary TRP intake in relation to metformin response [98,99]. These studies do not establish cardiovascular efficacy, but they support the feasibility of TRP-focused interventions and provide a rationale for future cardiometabolic trials.

Future trials should be designed around pathway phenotypes rather than treating TRP metabolism as a uniform target. For example, patients with HFpEF and reduced microbiome-derived IPA may be candidates for IPA supplementation or microbiome-directed interventions, whereas patients with impaired NAD^+^ metabolism may be better suited for NAD^+^-restoring strategies. Individuals with high KTR together with increased 3-HK, QA, or 3-HK/XA may represent a subgroup in which KMO/3-HK/QA-branch modulation could be explored. However, these examples remain hypotheses and should be tested in carefully phenotyped clinical cohorts before therapeutic conclusions are drawn. Trial endpoints should include not only clinical outcomes, but also mechanistic readouts such as endothelial function, vascular inflammation, myocardial energetics, exercise capacity, insulin sensitivity, skeletal muscle function, and tissue remodeling. Temporal profiling will be essential for interpreting such trials. Serial measurements can help determine whether TRP metabolic changes precede disease progression, reflect acute inflammatory injury, represent compensatory repair, or change in response to therapy. In MI, repeated sampling could clarify whether early kynurenine pathway activation contributes to injury and adverse remodeling or participates in repair. In HFpEF, longitudinal profiling could determine whether changes in KTR, IPA, NAD^+^-linked metabolites, or KMO-branch metabolites precede functional decline or track improvement after weight loss, exercise training, dietary intervention, microbiome modulation, or pharmacological therapy.

### 5.4. Future Directions

Several issues must be resolved before TRP metabolism can be translated into precision cardiometabolic medicine. First, most human studies rely on circulating metabolites, which do not define cellular sources or tissue-level pathway flux. This is particularly important because TRP metabolism may differ across vascular lesions, myocardium, adipose tissue, skeletal muscle, liver, kidney, immune cells, and gut compartments. Second, many available studies remain associative and cannot determine whether altered TRP metabolism is causal, compensatory, or simply a marker of inflammation and disease severity. Third, TRP metabolites are influenced by diet, renal function, age, sex, medication use, microbiome composition, and systemic inflammatory burden, all of which may confound interpretation.

Future studies should therefore combine circulating metabolomics with experimental systems that more closely reflect human cardiometabolic physiology. Although mouse models have been essential for identifying candidate mechanisms, they do not fully reproduce human cardiovascular structure, lipid metabolism, renal–cardiac interactions, immune responses, or microbiome complexity. Large-animal models, including porcine or canine models of MI, pressure overload, metabolic syndrome, or HFpEF-like disease, may provide an important bridge between small-animal studies and clinical translation. These models could help determine whether branch-specific TRP metabolite signatures observed in humans correspond to tissue-level pathway activity, myocardial energetics, vascular inflammation, plaque biology, and therapeutic response. In parallel, cell-specific and spatially resolved approaches will be needed to define where TRP metabolites are produced, consumed, and functionally active. Single-cell transcriptomics, spatial metabolomics, isotope-tracing studies, organ-specific sampling, and multi-omics integration may help identify the cellular sources and local consequences of altered TRP metabolism in cardiometabolic tissues. This is important because recent work in other fields has emphasized that TRP metabolism may occur in spatially restricted metabolic niches [100], where local TRP depletion, KYN accumulation, and downstream metabolite signaling differ across cell types and tissue regions [100]. These approaches are particularly relevant for heterogeneous syndromes such as HFpEF, where TRP metabolism may connect systemic inflammation, microbiome dysfunction, impaired myocardial energetics, and functional limitation.

Overall, the therapeutic relevance of TRP metabolism will depend on identifying which pathway branch is engaged, in which tissue, at what disease stage, and with what functional consequence. Addressing these questions will determine whether TRP metabolism can move beyond a biomarker of cardiometabolic risk toward a guide for pathway-informed therapeutic intervention.

## 6. Conclusions

TRP metabolism has emerged as an important metabolic interface linking inflammation, immune regulation, oxidative stress, endothelial function, and cellular energetics in cardiometabolic diseases. Although activation of the kynurenine pathway is consistently observed across obesity, T2D, atherosclerosis, MI, and HFpEF, the evidence reviewed here indicates that its biological meaning cannot be inferred from pathway activation alone. Instead, cardiometabolic outcomes appear to depend on how TRP-derived flux is distributed across downstream branches, which metabolites accumulate, and in which tissue or disease context these changes occur.

A central implication of this framework is that kynurenine pathway metabolites may exert divergent, and sometimes opposing, effects. KA-linked signaling may support anti-inflammatory, endothelial-protective, metabolic, or cardioprotective responses in selected settings, whereas KYN–AHR signaling and 3-HK-dependent redox stress may contribute to inflammation, oxidative injury, and tissue dysfunction. Similarly, QA may support NAD^+^ biosynthesis when downstream conversion is efficient, but inefficient QA-to-NAD^+^ flux may reflect impaired metabolic resolution under chronic inflammatory or oxidative stress. Microbiome-derived indole metabolites add another layer of regulation by linking diet, gut barrier integrity, immune signaling, and myocardial energetics.

These observations have direct translational relevance. The KTR remains a useful entry-point marker of upstream TRP-to-KYN conversion, but it is not disease-specific and does not define downstream pathway routing. More informative clinical interpretation will likely require metabolite panels that capture KA, KMO/3-HK/QA, 3-HAA-, microbiome-derived indole-, and NAD^+^-linked pathway activity. Likewise, therapeutic approaches should move beyond broad upstream pathway inhibition and instead consider disease stage, tissue compartment, and branch-specific metabolite profiles. Strategies that restore beneficial metabolite signaling, redirect maladaptive flux, support NAD^+^ metabolism, or modulate microbiome-derived indole pathways may be more appropriate than uniform suppression of TRP catabolism.

Despite substantial progress, important gaps remain. Most human studies are associative and rely on circulating metabolites, which cannot fully define cellular sources, tissue-level flux, or causal mechanisms. Future studies should integrate longitudinal temporal profiling, spatial and cell-specific analyses, isotope-tracing approaches, and large-animal models that better approximate human cardiometabolic physiology. Combining these tools with clinical phenotyping, imaging, inflammatory markers, renal function, microbiome data, and metabolite panels will be essential to determine whether altered TRP metabolism is a driver, compensatory response, or biomarker of disease progression. Such work will be necessary to translate TRP metabolism from a marker of cardiometabolic risk into a pathway-informed guide for precision therapeutic intervention.

## Figures and Tables

**Table 1:** **Translational opportunities and limitations for tryptophan metabolism in cardiometabolic diseases.** This table summarizes biomarker, therapeutic, and translational strategies related to tryptophan (TRP) metabolism, with emphasis on the kynurenine pathway and its downstream branches. The kynurenine-to-tryptophan ratio (KTR) reflects upstream TRP-to-kynurenine (KYN) conversion, whereas downstream metabolite panels may provide more branch-specific information, including kynurenic acid (KA)-linked regulatory signaling, kynurenine 3-monooxygenase (KMO)/3-hydroxykynurenine (3-HK)/quinolinic acid (QA)-linked redox activity, 3-hydroxyanthranilic acid (3-HAA)-related lipid and inflammasome regulation, nicotinamide adenine dinucleotide (NAD^+^)-linked metabolic resolution, and microbiome-derived indole pathways. Therapeutic translation requires disease- and tissue-context-specific interpretation because broad pathway inhibition may disrupt protective immune, vascular, or metabolic functions. AHR, aryl hydrocarbon receptor; DOX, doxorubicin; HF, heart failure; HFpEF, heart failure with preserved ejection fraction; IDO1, indoleamine 2,3-dioxygenase 1; IL, interleukin; IPA, indole-3-propionic acid; MI, myocardial infarction; oxLDL, oxidized low-density lipoprotein; SIRT3, sirtuin 3; T2D, type 2 diabetes; TDO, tryptophan 2,3-dioxygenase; XA, xanthurenic acid.

Approach	Rationale	Current Evidence	Translational Considerations
**KTR as an entry-point biomarker**	Reflects upstream TRP-to-KYN conversion and systemic kynurenine pathway activation.	Elevated KTR has been associated with atherosclerotic burden, coronary events, MI risk, cardiovascular mortality, HF outcomes, and impaired functional capacity [21,35,38,41,46,50,52].	Not disease-specific and does not identify tissue source or downstream branch routing. Best used for risk stratification or pathway monitoring within defined clinical contexts.
**Downstream metabolite panels**	Capture branch-specific pathway biology beyond KTR alone.	KA/QA and KA/3-HK ratios have been reported in T2D; altered KA/3-HAA balance is linked to symptomatic carotid disease; KYN, 3-HK, QA, KTR, and 3-HK/XA are associated with HF outcomes [14,19,49,50,52].	Panels may distinguish regulatory, redox-active, lipid/inflammasome-related, microbiome-derived, or NAD^+^-linked profiles. Requires assay standardization and adjustment for diet, renal function, inflammation, medications, and microbiome variation.
**IDO1/TDO inhibition**	May reduce upstream TRP catabolism and KYN generation in selected contexts.	IDO1 inhibitors such as epacadostat and navoximod and dual IDO1/TDO inhibitors such as M4112 have been tested mainly in oncology; epacadostat failed to improve outcomes in the phase III ECHO-301/KEYNOTE-252 melanoma trial [94,95,96,97].	No established cardiometabolic indication. Broad upstream inhibition may be harmful or ineffective because IDO1-derived metabolites can support immune tolerance, vascular protection, 3-HAA-associated anti-inflammatory effects, and QA-dependent NAD^+^ synthesis [23,24,26].
**KMO/3-HK/QA-branch modulation**	May reduce redox-active metabolite accumulation and oxidative injury when this branch is excessive.	3-HK promotes NADPH oxidase-dependent ROS generation, endothelial apoptosis, and dysfunction; HF studies link 3-HK, QA, and 3-HK/XA with adverse outcomes [6,50].	Should not be approached as simple KMO-branch suppression because the same branch also generates 3-HAA, which may be protective. Requires branch-specific metabolite profiling before intervention.
**NAD^+^ restoration or QA-to-NAD^+^ flux support**	Targets impaired metabolic resolution and mitochondrial energetics downstream of QA.	In DOX-induced cardiotoxicity models, impaired QA-to-NAD^+^ conversion was linked to reduced NAD^+^ and myocardial injury, while restoring this flux conferred cardioprotection [77].	May be more appropriate when NAD^+^-linked downstream flux is impaired rather than when upstream KYN activation alone is present. Should be paired with NAD^+^-related metabolite measurements.
**KA-enhancing or KA-mimetic strategies**	Support KA-dependent regulatory signaling through GPR35, AHR, or PPARδ/HO-1.	KA–GPR35 signaling supports adipose energy expenditure and anti-inflammatory programs; KA–PPARδ/HO-1 protects endothelial cells; KA–AHR reduces inflammatory macrophage signaling; KA has shown cardioprotective effects in ischemia–reperfusion models [18,19,55,56,75,76].	Effects are receptor, tissue, and disease-context dependent. Human cardiometabolic efficacy and optimal delivery remain unproven.
**3-HAA-related strategies**	May exploit anti-inflammatory and lipid-modulatory effects of 3-HAA.	3-HAA suppresses inflammasome activation, IL-1β production, SREBP-2/ApoB signaling, LDL oxidation, oxLDL uptake, and atherosclerotic lesion development in experimental models [20,22,29].	Evidence remains largely preclinical. Safety, dose, tissue specificity, and physiological relevance require further studies.
**Microbiome-derived indole strategies**	Restore beneficial TRP-derived indole signaling through AHR–IL-22 or IPA–AHR–SIRT3–NAD^+^ pathways.	IDO1 deficiency shifted intestinal TRP metabolism toward indole derivatives and improved gut barrier/metabolic phenotypes in obesity models; IPA supplementation improved myocardial energetics, fibrosis, inflammation, and diastolic dysfunction in HFpEF models [8,15].	Potential approaches include diet, prebiotics, probiotics, postbiotics, or metabolite supplementation. Translation requires attention to microbiome variability, bioavailability, dose, safety, and disease stage.
**Temporal profiling in clinical studies**	Defines whether pathway changes precede disease, reflect acute injury, mark repair, or respond to treatment.	Serial profiling may clarify TRP metabolic changes after MI, during HFpEF progression, or after weight loss, exercise, dietary intervention, microbiome modulation, or pharmacological therapy.	Needed to distinguish causal mechanisms from compensatory responses or markers of disease severity. Should be incorporated into future pathway-informed trials.
**Large-animal and spatial/cell-specific models**	Bridge the gap between small-animal mechanisms and human cardiometabolic disease.	Current evidence is largely based on circulating metabolites, cell studies, and mouse models. Large-animal models may better capture human cardiovascular structure, lipid metabolism, renal–cardiac interactions, immune responses, and microbiome complexity.	Porcine or canine models of MI, pressure overload, metabolic syndrome, or HFpEF-like disease, combined with isotope tracing, spatial metabolomics, and single-cell approaches, may help define tissue-level flux and therapeutic response.

## Data Availability

No new data were created or analyzed in this study. Data sharing is not applicable to this article.

## Abbreviations

1-MT	1-methyl-tryptophan
3,4-DAA	3,4-dimethoxycinnamoyl anthranilic acid
3-HAA	3-hydroxyanthranilic acid
3-HAO	3-hydroxyanthranilate dioxygenase
3-HK	3-hydroxykynurenine
AA	Anthranilic acid
ACMSD	α-amino-β-carboxy-muconate-semialdehyde decarboxylase
AHR	Aryl hydrocarbon receptor
AHRR	Aryl hydrocarbon receptor repressor
AIP	Aryl hydrocarbon receptor-interacting protein
AKT	Protein kinase B
ALAAD	Aromatic L-amino acid decarboxylase
AMPK	AMP-activated protein kinase
Ang II	Angiotensin II
ANG2	Angiopoietin-2
ApoB	Apolipoprotein B
ApoB100	Apolipoprotein B100
ApoE−/−	Apolipoprotein E-deficient
ARNT	Aryl hydrocarbon receptor nuclear translocator
ATP	Adenosine triphosphate
AUC	Area under the curve
BMI	Body mass index
C/EBPα	CCAAT/enhancer-binding protein alpha
CAD	Coronary artery disease
CCL2	C-C motif chemokine ligand 2
CCL5	C-C motif chemokine ligand 5
CCR7	C-C chemokine receptor 7
CD3	Cluster of differentiation 3
CD14	Cluster of differentiation 14
CD68	Cluster of differentiation 68
CREB	cAMP response element-binding protein
CRP	C-reactive protein
CTLA4	Cytotoxic T-lymphocyte-associated protein 4
CYP1A1	Cytochrome P450 1A1
CYP1A2	Cytochrome P450 1A2
CYP1B1	Cytochrome P450 1B1
c-Maf	Cellular musculoaponeurotic fibrosarcoma
c-Src	Proto-oncogene tyrosine-protein kinase Src
DC	Dendritic cell
DOX	Doxorubicin
EGF	Epidermal growth factor
EGFR	Epidermal growth factor receptor
ERK	Extracellular signal-regulated kinase
FABP4	Fatty acid-binding protein 4
FoxP3	Forkhead box P3
GPR35	G protein-coupled receptor 35
GTPγS	Guanosine 5′-O-(3-thiotriphosphate)
HAAO	3-hydroxyanthranilic acid 3,4-dioxygenase
HF	Heart failure
HFpEF	Heart failure with preserved ejection fraction
HFrEF	Heart failure with reduced ejection fraction
HFD	High-fat diet
HIF	Hypoxia-inducible factor
HIF1A	Hypoxia-inducible factor 1-alpha
HMG-CoA	3-hydroxy-3-methylglutaryl-coenzyme A
HO-1	Heme oxygenase-1
HSP90	Heat shock protein 90
HUVECs	Human umbilical vein endothelial cells
IAA	Indole-3-acetic acid
IAld	Indole-3-aldehyde
IDO	Indoleamine 2,3-dioxygenase
IDO1	Indoleamine 2,3-dioxygenase 1
IDO2	Indoleamine 2,3-dioxygenase 2
IFN-γ	Interferon gamma
IL-1β	Interleukin-1 beta
IL-6	Interleukin-6
IL-10	Interleukin-10
IL-17	Interleukin-17
IL-17A/F	Interleukin-17A/F
IL-22	Interleukin-22
IPA	Indole-3-propionic acid
JAK–STAT	Janus kinase–signal transducer and activator of transcription
KA	Kynurenic acid
KAT	Kynurenine aminotransferase
KAT1	Kynurenine aminotransferase 1
KAT2	Kynurenine aminotransferase 2
KLF6	Kruppel-like factor 6
KMO	Kynurenine 3-monooxygenase
KTR	Kynurenine-to-tryptophan ratio
KYN	Kynurenine
KYNs	Kynurenines
KYNU	Kynureninase
LC–MS/MS	Liquid chromatography–tandem mass spectrometry
LDL	Low-density lipoprotein
Ldlr−/−	Low-density lipoprotein receptor-deficient
LPS	Lipopolysaccharide
LV	Left ventricular
Ly6G	Lymphocyte antigen 6 complex locus G6
MAPK	Mitogen-activated protein kinase
MI	Myocardial infarction
mTOR	Mechanistic target of rapamycin
NAD^+^	Nicotinamide adenine dinucleotide
NADPH	Nicotinamide adenine dinucleotide phosphate
NAMPT	Nicotinamide phosphoribosyltransferase
NF-κB	Nuclear factor kappa B
NMDA	N-methyl-D-aspartate
NMN	Nicotinamide mononucleotide
NNMT	Nicotinamide N-methyltransferase
NO	Nitric oxide
NR	Nicotinamide riboside
NT-proBNP	N-terminal pro-B-type natriuretic peptide
oxLDL	Oxidized low-density lipoprotein
p23	Prostaglandin E synthase 3
p38 MAPK	p38 mitogen-activated protein kinase
p47phox	NADPH oxidase cytosolic subunit p47phox
p67phox	NADPH oxidase cytosolic subunit p67phox
p-ACCSer79	Phosphorylated acetyl-CoA carboxylase at Ser79
p-AKTSer473	Phosphorylated protein kinase B at Ser473
PBMCs	Peripheral blood mononuclear cells
pDC	Plasmacytoid dendritic cell
PDGFB	Platelet-derived growth factor subunit B
PGC-1α1	Peroxisome proliferator-activated receptor gamma coactivator 1-alpha 1
PLP	Pyridoxal 5′-phosphate
PPARδ	Peroxisome proliferator-activated receptor delta
PPARγ	Peroxisome proliferator-activated receptor gamma
p-HSLSer660	Phosphorylated hormone-sensitive lipase at Ser660
PTEN	Phosphatase and tensin homolog
QA	Quinolinic acid
QPRT	Quinolinate phosphoribosyltransferase
Rb	Retinoblastoma protein
Reg3b	Regenerating islet-derived protein 3 beta
Reg3g	Regenerating islet-derived protein 3 gamma
RGS14	Regulator of G protein signaling 14
ROS	Reactive oxygen species
SICA-HF	Studies Investigating Co-morbidities Aggravating Heart Failure
SIRT1	Sirtuin 1
SIRT3	Sirtuin 3
SLC1A5	Solute carrier family 1 member 5
SLC7A5	Solute carrier family 7 member 5
SMC	Smooth muscle cell
SREBP-2	Sterol regulatory element-binding protein 2
STAT3	Signal transducer and activator of transcription 3
ST2	Suppression of tumorigenicity 2
STING	Stimulator of interferon genes
T2D	Type 2 diabetes
TCA cycle	Tricarboxylic acid cycle
TDO	Tryptophan 2,3-dioxygenase
TDO2	Tryptophan 2,3-dioxygenase 2
TF	Tissue factor
TGFβ2	Transforming growth factor beta 2
THP-1	Human monocytic leukemia cell line
TNF-α	Tumor necrosis factor alpha
TPH	Tryptophan hydroxylase
Treg	Regulatory T cell
TRP	Tryptophan
VCAM-1	Vascular cell adhesion molecule 1
XA	Xanthurenic acid
XAP2	Hepatitis B virus X-associated protein 2
XRE	Xenobiotic response element
β-AR	Beta-adrenergic receptor
αKG	Alpha-ketoglutarate
